# Prevalence of Chronic Pain and Associated Factors Among Nursing Students in Heilongjiang Province, China: A Cross‐Sectional Study

**DOI:** 10.1002/nop2.70685

**Published:** 2026-08-13

**Authors:** Siqi Liu, Yujin Xie, Zhengxi Chen, Xiwei Chen, Lei Cao, Yue Qin, Mei Yin, Yang Fu, Lei Shi

**Affiliations:** ^1^ The Fourth Affiliated Hospital of Harbin Medical University Harbin China; ^2^ West China Hospital of Sichuan University Chengdu China; ^3^ Beijing Rehabilitation Hospital, Capital Medical University Beijing China; ^4^ The Second Affiliated Hospital of Harbin Medical University Harbin China; ^5^ School of Health Management Guangzhou Medical University Guangzhou China; ^6^ Department of Nursing Heilongjiang Nursing College Harbin China; ^7^ The First Affiliated Hospital of Harbin Medical University Harbin China; ^8^ Department of Medical Ethics, School of Humanities Harbin Medical University Harbin China; ^9^ Philosophy and Social Sciences Key Laboratory of Guangdong Higher Education Institutes for Health Governance Based on Big Data Utilization Guangzhou Medical University Guangzhou China

**Keywords:** associated factors, chronic pain, nursing students, public health, sleep quality

## Abstract

**Aim:**

This study explored the prevalence and potential factors influencing chronic pain and provided increased theoretical and practical support for future studies on nursing students from a novel perspective.

**Design:**

This study applied a cross‐sectional study design.

**Methods:**

A cross‐sectional survey with 1825 nursing students was conducted at Heilongjiang Nursing College in Heilongjiang province of northern China, recruited using cluster sampling. Multivariate logistic regression analyses explored the factors associated with chronic pain among nursing students.

**Results:**

The final study sample included 1802 nursing students and the prevalence of chronic pain among first‐year nursing students was 59.2%. The top five sites of body pain in the past year were the neck, head, lower back, shoulder, and legs. Sex, family history of chronic pain, alcohol consumption, and sleep quality were significantly associated with chronic pain.

**Conclusion:**

Chronic pain was common among this sample of nursing students. Early assessment and intervention at different pain sites are necessary to reduce the potential negative effects of pain. Developing targeted inventions to improve chronic pain and enhance students' quality of life is crucial.

**Implications for the Profession and/or Patient Care:**

Nursing students may suffer from chronic pain due to many potentially influential factors. In the process of daily management, schools or related personnel can take effective measures to intervene in these factors, thus reducing the prevalence of chronic pain and improving the quality of life.

**Patient or Public Contribution:**

No patient or public contribution.

## 1 Introduction

Pain is a serious global public health concern affecting individuals of all ages and professions (Yongjun et al. 2020; Kuehn 2018). The Global Burden of Disease 2017 has reported that low back pain and headache disorders are the leading causes of years lost to disability (GBD 2017 Disease and Injury Incidence and Prevalence Collaborators 2018). The International Association for the Study of Pain defines pain as an unpleasant sensory and emotional experience associated with or resembling actual or potential tissue damage (Raja et al. 2020). Pain is typically classified as acute or chronic, where chronic pain refers to pain that persists for more than three months and may be continuous or recurrent (Steglitz et al. 2012; Treede et al. 2019).

The Institute of Medicine has indicated that chronic pain affects millions of American adults—greater than the total incidence of heart disease, cancer, and diabetes combined (Steglitz et al. 2012). The prevalence of chronic pain among adults in the United States is estimated to be 20.4% and varies between 35.0% and 51.3% in the United Kingdom (Dahlhamer et al. 2018; Moffett et al. 1993). In China, chronic pain appears to be increasingly prevalent as the pressure on survival and academic demands increases considerably among young people (Yongjun et al. 2020; Zhang et al. 2015). Previous research has found that the rate of chronic pain has reached 31.5% (Yongjun et al. 2020). In Shanghai, China, the rate of low back pain among high school students is 32.8%, and the prevalence rates of headache (30.3%) and abdominal pain (20.9%) are far from low (Zhang et al. 2015), showing that the chronic pain trend is becoming increasingly younger.

As a special medical service group, nursing staff work under distinctive conditions, including long working hours, heavy work‐related stress, irregular rest times, and highly repetitive postures, such as stooping and crouching, increasing the probability of chronic pain. Nursing students, being the mainstay of the future nursing talent pipeline, must spend much time and energy acquiring professional knowledge and practical skills. Over several years in school, numerous nursing students are employed to accomplish learning tasks, ranging from sedentary learning to standing and repetitive stooping during clinical training, potentially jeopardising their physical well‐being and increasing their risk of chronic pain (Moffett et al. 1993; Cedercreutz et al. 1987; Grasdalsmoen et al. 2020). Therefore, emphasising health education for nursing students to develop healthy behavioural habits is necessary.

Owing to the challenging working conditions experienced by nursing students, identifying potential risk factors and providing preventive measures to avoid or reduce the incidence of chronic pain is essential. Although several epidemiologic studies have investigated chronic pain in university students, most research has focused on medical or undergraduate students rather than nursing students, and reported the prevalence of chronic pain in the student population (Grasdalsmoen et al. 2020; Chan et al. 2020; Alhowimel et al. 2022; Dighriri et al. 2019; Kim et al. 2021; Hamaoka et al. 2022; Serbic et al. 2023; Algarni et al. 2017; Weleslassie et al. 2020; Wang et al. 2023; Kanchanomai et al. 2011; Taha et al. 2023; Behera et al. 2020). The prevalence of chronic pain among undergraduate students in Norway, Japan, and South Korean was found to be 54%, 30.0%, and 7.8%, respectively (Grasdalsmoen et al. 2020; Kim et al. 2021; Hamaoka et al. 2022). The sites and prevalence of chronic pain greatly vary among students with different majors because of differences in their programs' content, learning time, and stress (Chan et al. 2020). Therefore, this study, conducted with a sample of first‐year nursing students at Heilongjiang Nursing College in Heilongjiang province of northern China, aimed to comprehensively categorise pain sites and to provide a detailed analysis and discussion of the potential factors influencing chronic pain, such as family background and behaviours concerning chronic pain. The study aimed to provide increased theoretical and practical support for future studies on nursing personnel from a novel perspective.

## 2 Materials and Methods

### 2.1 Design and Sample

**FIGURE 1:**
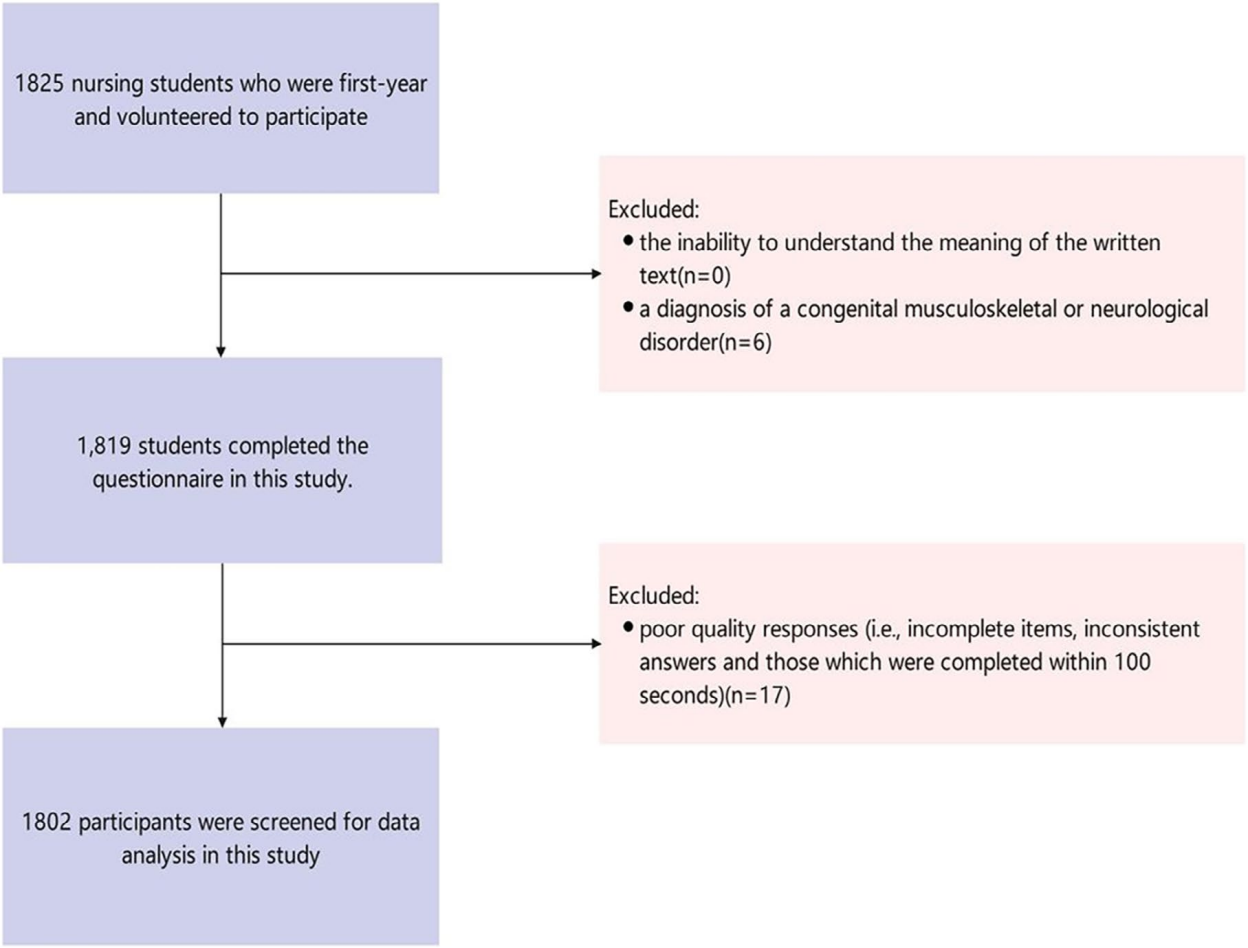
Flowchart of participant selection.

A cross‐sectional survey was conducted at Heilongjiang Nursing College, a vocational college in Heilongjiang Province, northern China, that specialises in nursing education, between June 2023 and October 2023 using a cluster sampling method. We distributed and collected the corresponding questionnaires through the internet. To ensure that there were no missed answers, all questions had to be answered. Each IP address was limited to one response to ensure the quality of the survey. The researchers sent the questionnaire to the participants through We Chat, and the participants filled out the questionnaire on their mobile phones. A total of 1825 questionnaires distributed, 1802 were deemed valid, with an effective recovery rate of 98.74%. Individuals were included in the study if they (1) were first‐year and (2) volunteered to participate. The exclusion criteria included: (1) poor quality responses (i.e., incomplete items, inconsistent answers and those which were completed within 100 s), (2) the inability to understand the meaning of the written text, and (3) a diagnosis of a congenital musculoskeletal or neurological disorder (Figure 1). The detailed recruitment process, including channels, timeline, invitation wording, participation rates, exclusion criteria, and incentives, is provided in the Appendix.

### 2.2 Data Collection

The questionnaire comprised three parts. The first part included demographic items, such as sex, age, height, body weight, and Family residence. The relevant assignment rules are detailed in Table 1. In the second section, students responded to items regarding the presence of chronic pain using multiple‐choice questions. These items covered various aspects, ranging from whether they were currently experiencing pain to the location and self‐perceived intensity of the pain. Example questions included: “Have you had persistent or recurrent pain lasting more than three months in the past year?”, “What parts of your body are in pain?”, and “How would you rate your pain intensity?” (assessed using the Numerical Rating Scale). The third part collected information on participants' lifestyles that may be associated with pain (i.e., smoking, alcohol consumption, frequency of ordering takeaway food per week, frequency of physical exercise per week). These items were intended to investigate and analyse the prevalence, site, and potential influences of pain on students' lives. Although questionnaires containing more questions can help identify additional risk factors, the items that are asked later on the survey have the risk of being lower quality data, especially if they are in an open format (Galesic and Bosnjak 2009). Therefore, we aimed to keep the questionnaire as short as possible and used multiple‐choice responses as the primary survey method. The questionnaire employed in this study consisted primarily of factual items concerning demographic characteristics and individual behavioural habits, and did not include multi‐item scales; consequently, internal consistency reliability was not assessed. A pilot test of the questionnaire was conducted among 50 participants recruited from the target population. Based on participant feedback, ambiguous questions were revised to improve clarity and usability. No major technical issues with the online survey platform were identified. Pilot data were not included in the final analysis.

**TABLE 1:** Description and assignment of study variables.

Variable category	Variable name	Assignment
Dependent variable	Chronic pain	1 = Yes, 2 = No
Personal characteristic	Gender	1 = Female, 2 = Male
	BMI	1 = ≤ 18.4, 2 = 18.5–23.9, 3 = 24–27.9, 4 = ≥ 28
	Academic stress	1 = Extremely low, 2 = Very low, 3 = Fair, 4 = Very high, 5 = Extremely high
Family background	Family residence	1 = Urban, 2 = Suburb
	Monthly living expenses (yuan)	1 = < 500, 2 = 500–1000, 3 = 1000–1500, 4 = 1500–2000, 5 = > 2000
	Family history of chronic pain	1 = Yes, 2 = No
Behavioural characteristics	Alcohol consumption	1 = Yes, 2 = No
	Smoking habit	1 = Yes, 2 = No
	Frequency of ordering takeaway food per week	1 = > 0, 2 = 1–3, 3 = 4–7, 4 = 8–15, 5 = > 15
	Duration of electronic products per day (hours)	1 = < 2, 2 = 2–5, 3 = 5–8, 4 = 8–12, 5 = > 12
	Frequency of physical exercise per week	1 = 0, 2 = 1–2, 3 = 3–4, 4 = 5–6, 5 = > 7
	Duration of learning per day (hours)	1 = < 2, 2 = 2–5, 3 = 5–8, 4 = 8–12, 5 = > 12
	Sleep quality	1 = Very poor, 2 = Poor, 3 = Fair, 4 = Good, 5 = Excellent

### 2.3 Data Analysis

This study followed the Strengthening the Reporting of Observational Studies in Epidemiology (STROBE) reporting guideline. The STROBE checklist for cross‐sectional studies was used to ensure complete and transparent reporting of the study design, data collection, statistical analysis, and interpretation of results. Preliminary analysis showed that the cluster variable did not significantly affect the outcome, likely due to large within‐cluster heterogeneity and/or small between‐cluster variation. Therefore, no cluster adjustment methods (e.g., cluster‐robust standard errors or multilevel modelling) were applied in the main analysis. Categorical variables were expressed as numbers and percentages, and chi‐square tests were used to compare the in‐pain and not‐in‐pain student groups. In the chi‐square tests, the presence of pain was used as a bivariate categorical variable to calculate the *p*‐values for the comparisons. The analyses and *p*‐values were two‐tailed, and statistical significance was set at *p* < 0.05. The Bonferroni correction was applied to control the Type I error. Multivariate logistic regression analyses were used to further examine the potential factors associated with the risk of pain, and odds ratios (ORs) and 95% confidence intervals (CIs) were calculated. The following independent variables were entered into the multivariable logistic regression model: gender, age, BMI, academic stress, family residence, monthly living expenses (yuan), family history of chronic pain, alcohol consumption, smoking habit, weekly frequency of ordering takeaway food, daily duration of electronic product use (hours), weekly frequency of physical exercise, daily duration of learning (hours), and sleep quality (Chen et al. 2025; Chunming et al. 2013; Nadeem et al. 2017; Liu et al. 2023; Wong et al. 2022; Iqbal et al. 2015; Luo et al. 2021; Grasdalsmoen et al. 2020). These variables were all preselected based on established literature and theoretical considerations, and were not subjected to significance‐based screening (e.g., stepwise selection or *p*‐value filtering from univariate analyses). In this model, we included latent variables, with a significance level set at *p* < 0.05. All analyses were performed using SPSS Statistical Software version 25.0. To assess the robustness of the findings, sensitivity analyses were conducted. First, the sample sizes for all categorical variables were examined, and the results indicated that each group contained more than 50 cases, with no rare categories requiring consolidation. Subsequently, generalised linear models incorporating Huber‐White robust standard errors were employed to re‐estimate the associations between various factors and chronic pain. This approach was used to correct for potential heteroscedasticity, thereby yielding more reliable standard errors and confidence intervals.

### 2.4 Ethical Considerations

All study procedures were approved by the Ethics Committee of the Beijing Rehabilitation Hospital, Capital Medical University (approval number: 2023bkky‐097; date: 2023‐06‐01) in accordance with the Declaration of Helsinki. All participants were informed of the purpose of this study and provided signed informed consent.

## 3 Results

### 3.1 Sociodemographic Characteristics of Participants

Table 2 presents the participants' sociodemographic characteristics. Of the 1802 subjects, most of them were women (*n* = 1411, 78.3%). More than half (*n* = 1014, 56.3%) of students' homes were in urban areas (*n* = 1014, 56.3%). The participants' monthly costs of living were concentrated at 1500 yuan and above (*n* = 609, 33.8%). The number of individuals who purchased takeout food more than eight times per week was 407 (23.6%).

**TABLE 2:** General characteristics of participants (*N* = 1802).

Variables	*n*	Percent (%)
Personal characteristic		
Gender		
Male	391	21.7
Female	1411	78.3
BMI (Chunming et al. 2013)		
≤ 18.4	468	26.0
18.5–23.9	958	53.2
24–27.9	281	15.5
≥ 28	95	5.3
Academic stress (Nadeem et al. 2017)		
Extremely low	183	10.2
Very low	160	8.8
Fair	1045	58.0
Very high	268	14.9
Extremely high	146	8.1
Family background		
Family residence (Liu et al. 2023)		
Urban	1014	56.3
Suburb	788	43.7
Monthly living expenses (yuan) (Liu et al. 2023)		
< 500	107	5.9
500–1000	285	15.8
1000–1500	595	33.1
1500–2000	609	33.8
> 2000	206	11.4
Family history of chronic pain (Wong et al. 2022)		
Yes	193	10.7
No	1609	89.3
Behavioral characteristics		
Alcohol consumption (Iqbal et al. 2015)		
Yes	204	11.3
No	1598	88.7
Smoking habit (Iqbal et al. 2015)		
Yes	190	10.5
No	1612	89.5
Frequency of ordering takeaway food per week (Luo et al. 2021)		
0	566	31.4
1–3	509	28.2
4–7	320	17.8
8–15	130	8.2
> 15	277	15.4
Duration of electronic products per day (hours) (Chen et al. 2025)		
< 2	180	10.0
2–5	441	24.5
5–8	711	39.5
8–12	288	16.0
> 12	182	10.0
Frequency of physical exercise per week (Grasdalsmoen et al. 2020)		
0	182	10.1
1–2	767	42.6
3–4	553	30.7
5–6	124	6.9
> 7	176	9.7
Duration of learning per day (hours)		
< 2	259	14.4
2–5	479	26.6
5–8	738	41.0
8–12	236	13.0
> 12	90	5.0
Sleep quality (Liu et al. 2023)		
Very poor	195	10.8
Poor	239	13.3
Fair	887	49.2
Good	348	19.3
Excellent	133	7.4

### 3.2 Prevalence and Common Sites of Chronic Pain

Table 3 shows the prevalence and sites of chronic pain in the past year reported by the nursing students. Of the 1802 participants, 1066 students (59.2%) experienced chronic pain. The top five sites of body pain in the past year were: neck (*n* = 781, 43.3%), head (*n* = 668, 37.1%), lower back (*n* = 666, 37.0%), shoulder (*n* = 603, 33.5%), and legs (*n* = 550, 30.5%).

**TABLE 3:** Prevalence and site of chronic pain among nursing students.

Variables	*n*	Percent (%)
Chronic pain		
Yes	1066	59.2
No	736	40.8
Pain sites		
Head	668	37.1
Neck	781	43.3
Shoulder	603	33.5
Arms	388	21.5
Chest	296	16.4
Abdomen	387	21.5
Low back	666	37.0
Back	444	24.6
Legs	550	30.5
Feet	387	21.5
Other	213	11.8

### 3.3 Differences in Physical Pain Among Participants by Sociodemographic Characteristics

The pain rates among nursing students significantly differed (*p* < 0.05) by sex, academic stress, family history of chronic pain, alcohol consumption, smoking, frequency of ordering takeout food per week, duration of electronic products per day, and quality of sleep (Table 4).

**TABLE 4:** Differences in physical pain among participants with different sociodemographic characteristics (*N* = 1802).

Sociodemographic characteristics	Chronic pain		*p* for trend
	Yes (*n*, %)	No (*n*, %)	
Personal characteristic			
Gender			< 0.001
Male	195 (49.9)	196 (50.1)	
Female	871 (61.7)	540 (38.3)	
BMI			0.160
< 18.5	269 (57.5)	199 (42.5)	
18.5–24	570 (59.5)	398 (40.5)	
24–28	160 (56.9)	121 (43.1)	
≥ 28	67 (70.5)	28 (29.5)	
Academic stress			< 0.001
Extremely low	99 (54.1)	84 (45.9)	
Very low	86 (53.8)	74 (46.2)	
Fair	587 (56.2)	458 (43.8)	
Very high	186 (69.4)	82 (30.6)	
Extremely high	108 (74.0)	38 (26.0)	
Family background			
Family residence			0.988
Urban	600 (59.2)	414 (40.8)	
Suburb	466 (59.1)	322 (40.9)	
Monthly living expenses (yuan)			0.057
< 500	58 (54.2)	49 (45.8)	
500–1000	194 (68.1)	91 (31.9)	
1000–1500	355 (59.7)	240 (40.3)	
1500–2000	341 (56.0)	268 (44.0)	
> 2000	118 (57.3)	88 (42.7)	
Family history of chronic pain			< 0.001
Yes	168 (87.0)	25 (13.0)	
No	898 (55.8)	711 (44.2)	
Behavioral characteristics			
Alcohol consumption			< 0.001
Yes	145 (71.1)	59 (28.9)	
No	921 (57.6)	677 (42.4)	
Smoking habit			0.010
Yes	129 (67.9)	61 (32.1)	
No	937 (58.1)	675 (41.9)	
Frequency of ordering takeaway food per week			< 0.001
0	298 (52.7)	268 (47.3)	
1–3	302 (59.3)	207 (40.7)	
4–7	197 (61.6)	123 (38.4)	
8–15	86 (66.2)	44 (33.8)	
> 15	183 (66.1)	94 (33.9)	
Duration of electronic products per day (hours)			< 0.001
< 2	94 (52.2)	86 (47.8)	
2–5	236 (53.5)	205 (46.5)	
5–8	418 (58.8)	293 (41.2)	
8–12	192 (66.7)	96 (33.3)	
> 12	126 (69.2)	56 (30.8)	
Frequency of physical exercise per week			0.124
0	116 (63.7)	66 (36.3)	
1–2	456 (59.5)	311 (40.5)	
3–4	328 (59.3)	225 (40.7)	
5–6	66 (53.2)	58 (46.8)	
> 7	100 (56.8)	76 (43.2)	
Duration of learning per day (hours)			0.937
< 2	154 (59.5)	105 (40.5)	
2–5	288 (60.1)	191 (39.9)	
5–8	426 (57.7)	312 (42.3)	
8–12	142 (60.2)	94 (39.8)	
> 12	56 (62.2)	34 (37.8)	
Sleep quality			< 0.001
Very poor	144 (73.8)	51 (26.2)	
Poor	182 (76.2)	57 (23.8)	
Fair	504 (56.8)	383 (43.2)	
Good	178 (51.1)	170 (48.9)	
Excellent	58 (43.6)	75 (56.4)	

### 3.4 Binary Logistic Regression Analysis of Chronic Pain

In the regression analysis, age was included in the model as a continuous variable. For the categorical variables, the following reference groups were used: “Female” for gender, “≤ 18.4” for BMI, “Extremely low” for academic stress, “Urban” for family residence, “< 500” for monthly living expenses (yuan), “Yes” for family history of chronic pain, “Yes” for alcohol consumption, “Yes” for smoking habit, “0” for frequency of ordering takeaway food per week, “< 2” for duration of electronic products per day (hours), “0” for frequency of physical exercise per week, “< 2” for duration of learning per day (hours), and “Very poor” for sleep quality. All categorical variables were entered into the regression model as dummy variables, with the reference groups specified as above. Table 5 showed that personal characteristics (i.e., being female), family background (i.e., family history of chronic pain), and behavioural characteristics (i.e., alcohol consumption and sleep quality) were significantly associated with chronic pain. Men were at lower risk for pain compared with women (OR = 0.543, 95% CI: 0.417, 0.706). Family history of pain was a risk factor for the development of chronic pain; hence, nursing students without a family history of pain were at lower risk for experiencing pain (OR = 0.228 95% CI: 0.144, 0.361). In this study, sleep quality and academic pressure were initially entered into the model as continuous variables to test for linear trends. The results showed that neither variable was statistically significant (*p* > 0.05); therefore, they were retained in the final model as ordinal categorical variables. The Box‐Tidwell approach was employed to test the linearity of the logit for age, and the results indicated that the interaction term between age and its natural logarithm was not statistically significant (*p* > 0.05), suggesting that the linearity assumption for age was satisfied. The discriminative ability of the model was evaluated using the C‐statistic (i.e., the area under the receiver operating characteristic curve, AUC), which yielded a value of 0.695 (95% CI: 0.671–0.719), indicating moderate discriminative power. Influence diagnostics, including assessments of Cook's distance, leverage values, and standardised residuals, revealed no outliers exerting undue influence on the parameter estimates.

**TABLE 5:** The factors associated with chronic pain.

Variables	B	S.E.	Wald	*p*	OR	95% CI	VIF
Personal characteristic							
Gender							1.141
Female					Reference		
Male	−0.611	0.134	20.807	< 0.001	0.543	(0.417, 0.706)	
Age	−0.041	0.040	1.059	0.303	0.960	(0.888, 1.038)	1.030
BMI							1.009
≤ 18.4					Reference		
18.5–23.9	0.613	0.261	5.527	0.019	1.846	(1.107, 3.077)	
24–27.9	0.459	0.250	3.360	0.067	1.582	(0.969, 2.584)	
≥ 28	0.600	0.274	4.810	0.028	1.823	(1.066, 3.116)	
Academic stress							1.094
Extremely low					Reference		
Very low	0.463	0.269	2.967	0.085	1.589	(0.938, 2.692)	
Fair	0.730	0.282	6.688	0.010	2.075	(1.193, 3.608)	
Very high	0.684	0.238	8.275	0.004	1.982	(1.244, 3.159)	
Extremely high	0.160	0.265	0.362	0.547	1.173	(0.697, 1.974)	
Family background							
Family residence							1.088
Urban					Reference		
Suburb	−0.118	0.109	1.172	0.279	0.889	(0.718, 1.100)	
Monthly living expenses (yuan)							1.125
< 500					Reference		
500–1000	0.237	0.281	0.710	0.400	1.267	(0.730, 2.199)	
1000–1500	−0.676	0.214	9.979	0.002	0.509	(0.334, 0.774)	
1500–2000	−0.358	0.184	3.795	0.051	0.699	(0.488, 1.002)	
> 2000	−0.125	0.179	0.489	0.484	0.882	(0.622, 1.253)	
Family history of chronic pain							1.088
Yes					Reference		
No	−1.477	0.234	39.950	< 0.001	0.228	(0.144, 0.361)	
Behavioral characteristics							
Alcohol consumption							1.270
Yes					Reference		
No	−0.514	0.196	6.855	0.009	0.598	(0.407, 0.879)	
Smoking habit							1.351
Yes					Reference		
No	−0.173	0.207	0.700	0.403	0.841	(0.561, 1.262)	
Frequency of ordering takeaway food per week							1.110
0					Reference		
1–3	0.399	0.170	5.508	0.019	1.490	(1.068, 2.079)	
4–7	0.160	0.173	0.858	0.354	1.173	(0.837, 1.646)	
8–15	0.033	0.188	0.031	0.860	1.034	(0.715, 1.494)	
> 15	−0.016	0.242	0.004	0.947	0.984	(0.613, 1.580)	
Duration of electronic products per day (hours)							1.143
< 2					Reference		
2–5	0.612	0.248	6.064	0.014	1.843	(1.133, 2.999)	
5–8	0.467	0.217	4.653	0.031	1.596	(1.044, 2.440)	
8–12	0.195	0.204	0.915	0.339	1.215	(0.815, 1.813)	
> 12	−0.139	0.223	0.388	0.533	0.870	(0.562, 1.348)	
Frequency of physical exercise per week							1.099
0					Reference		
1–2	0.094	0.249	0.141	0.707	1.098	(0.674, 1.788)	
3–4	−0.025	0.196	0.017	0.897	0.975	(0.663, 1.432)	
5–6	−0.086	0.200	0.183	0.669	0.918	(0.620, 1.358)	
> 7	0.098	0.262	0.140	0.709	1.103	(0.660, 1.842)	
Duration of learning per day (hours)							1.078
< 2					Reference		
2–5	0.162	0.285	0.326	0.568	1.176	(0.674, 2.054)	
5–8	0.080	0.274	0.086	0.770	1.084	(0.633, 1.854)	
8–12	0.225	0.266	0.719	0.396	1.253	(0.744, 2.110)	
> 12	0.253	0.288	0.772	0.379	1.288	(0.732, 2.267)	
Sleep quality							1.096
Very poor					Reference		
Poor	−1.165	0.262	19.837	< 0.001	0.312	(0.187, 0.521)	
Fair	−1.485	0.252	34.590	< 0.001	0.227	(0.138, 0.372)	
Good	−0.607	0.208	8.514	0.004	0.545	(0.363, 0.819)	
Excellent	−0.460	0.223	4.240	0.039	0.631	(0.407, 0.978)	

To examine whether multicollinearity exists among the independent variables, the variance inflation factor (VIF) was calculated. The results show that the VIF values of all independent variables are well below the commonly used threshold of 5, indicating no serious multicollinearity issues among the predictors. Thus, the regression model results are stable and reliable.

After correction using robust standard errors, the measures of association (odds ratios) between each variable and chronic pain were consistent with those obtained in the primary analysis. The variables that were statistically significant in the primary analysis remained so, indicating that the findings of this study are relatively robust.

To assess the potential impact of the cluster sampling by grade level on the study results, we constructed an empty linear mixed model with grade as a random effect to calculate the intraclass correlation coefficient (ICC) for the dependent variable “chronic pain.” The results showed a between‐grade variance component of 0.000218 and a within‐student variance component of 0.241729, yielding an ICC of approximately 0.0009. This indicates that only about 0.09% of the total variance in chronic pain could be explained by differences across grades, while the vast majority (99.91%) of the variance originated from the individual student level.

Based on the ICC value and the average number of students per grade (*n* = 360), we further calculated the design effect (DEFF) as 1 + (*n* − 1) × ICC ≈1.321+ (*n* − 1) × ICC ≈1.32, which is substantially smaller than the empirical threshold of 2. These statistical findings demonstrate that the clustering effect at the grade level is very weak and has a negligible impact on the standard errors of the regression coefficients. Consequently, no statistical correction for cluster sampling is required in this study.

### 3.5 Hosmer‐Lemeshow Test Results

The chi‐square value was 7.980, *p*‐value was 0.435. This indicates that the model exhibits good fit, as the null hypothesis of adequate fit cannot be rejected at conventional significance levels (e.g., α = 0.05).

## 4 Discussion

This study aimed to determine the prevalence and sites of chronic pain among first‐year nursing students at Heilongjiang Nursing College in Heilongjiang province of northern China and to identify their associated factors as a foundation for future related research.

The current findings showed that the prevalence of chronic pain among nursing students over the past year was 58.90%, which is higher than that reported in Iran (30.2%) (Shaygan et al. 2022) and lower than that reported in Tokyo, Japan (79.2%), another Asian country (Kodama et al. 2021). These differences may be related to a lack of consistency in the definitions and criteria used to assess chronic pain across the studies (Steingrímsdóttir et al. 2017). Further, cultural factors, such as the study's setting, could be additional variables influencing these differences as culture differs from country to country. In some cultures, especially in Asia, pain is equivalent to stigma (Perugino et al. 2022). Thus, individuals may hide their physical pain because of the belief that speaking out about pain is a sign of weakness and may mask the illness severity and delay medical care, which prevents patients from receiving timely treatment and aggravates their distress, leading to an increased prevalence of chronic pain.

The prevalence of chronic pain is related to the educational environment. In China, economic disparity varies between regions, and the quality of the educational equipment in colleges and universities also varies. Poor bed comfort and inappropriate desk and chair heights may exacerbate differences in the prevalence of pain among nursing students in other countries. Further, the nursing students' learning intensity is high. As future nurses, nursing students may spend little time socialising and much time studying, decreasing the available time to go out with friends for exercise and relaxation. This pattern of behaviour could lead students to remain in chronic psychological stress and anxiety. Such time management strategies could increase the likelihood of psychosomatic disorders that contribute to chronic pain (Aaron et al. 2025).

We found that the six most common sites of pain in the past year for nurses were the neck (43.3%), head (37.1%), lower back (37.0%), shoulders (33.5%), legs (30.5%), and back (24.6%). Neck and lower back pain were more common than shoulder pain, consistent with previous studies. This difference in the site of pain may be related to the high intensity of nursing students' studies. Nursing students must stay late to learn and practice their skills during the theoretical phase of their studies, and they may also have night shifts in hospitals during the clinical phase. The lack of sleep and overwork are predisposing factors for headaches. Moreover, mental tension and stress in dealing with emergencies during clinical practicum are thought to trigger headaches. Simultaneously, nurses face physical demands, and nursing students are required to straighten their backs most of the time. This habit facilitates the muscles becoming stronger, enhancing the pulling and fixing effect on the bony structures and reducing the vertical extrusion on the intervertebral discs or other structures, which reduces the chances of herniation of the lumbar intervertebral discs and chronic pain (Du et al. 2025). Additionally, nursing students spend more time practicing clinical skills than medical students, who are equally burdened with coursework, meaning that nursing students change their posture rather than remain seated all the time. These shifts in posture can change the muscle force and relax the lower back muscle groups, relatively reducing the lower back muscle's long‐term tension state and the probability of insufficient oxygen supply by putting pressure on small blood vessels, accumulating metabolites, and stimulating inflammation (Jiang et al. 2025). Relief can only be relatively effective; thus, the overall picture of chronic low back pain among nursing students remains relatively unfavourable. Moreover, straightening of the lower back implies a relatively elevated head and neck position; therefore, students may press their heads lower and increase the angle of forward neck flexion. From the perspective of mechanical analysis, this also greatly increases the force acting on the cervical spine. Over time, prolonged head‐down work aggravates neck muscle spasms or chronic strain and may even cause the vertebrae to compress the nerve root, resulting in the generation of long‐term radiating pain of cervical spondylosis.

Data from Shanghai, in southern China, showed the overall prevalence rates for headache, neck and shoulder pain, and lower back pain were 30.3%, 41.1%, and 32.8%, respectively (Zhang et al. 2015), lower than the corresponding rates in this study (37.1% in the head, 43.3% in the neck, and 37.0% in the lower back). This north–south difference in prevalence may be related to climate. As the northernmost province in China, Heilongjiang Province has an average annual high temperature of 36°C and low temperatures that can reach −40°C. Long, cold winters with heavy snow reduce opportunities for people to walk, run, and relax outdoors. Simultaneously, a cold environment adversely affects the body's structures, such as the bones and joints, which are significantly associated with chronic pain (Farbu et al. 2019). However, the observed differences may be associated with differences in the participants' ages. The age range of the sample in Shanghai was 16–18 years (Zhang et al. 2015), while our sample had an age range of 16–28 years. Chronic pain involves the accumulation of time, and many patients experience chronic pain at certain stages of life, with the pain extending for a long period and even becoming increasingly severe with time. For example, intervertebral disk degeneration is the underlying cause of lumbar disk herniation in chronic pain. As age increases, the intervertebral disc gradually degenerates, and the water content of the annulus fibrosus and nucleus pulposus gradually decreases, losing elasticity and reducing its ability to resist external forces. Thus, the probability of chronic pain gradually increases with the accumulation of strain and external forces (Zhang et al. 2023).

This epidemiological survey yielded a higher prevalence of chronic pain than reported in other countries and regions with similar latitudes to Heilongjiang Province. The rate of chronic pain in Europe was 19% (Breivik et al. 2006). In the UK, a prevalence rate of 14.3% was reported by young adults aged 18–25 years (Fayaz et al. 2016). In Germany, the rate was 31.0% among adolescent students (Könning et al. 2021). In France, the prevalence of chronic neck and shoulder pain in France was 14.8% in women and 7.8% in men (Cassou et al. 2002). This diversity in prevalence is closely related to climate. Europe is surrounded by oceans, which have heat capacities that differ from land. When the temperature decreases, the sea gradually releases heat, causing gradual temperature changes. Moreover, some parts of Europe have temperate oceanic climates. The warm North Atlantic currents blow in with wind and moisture, changing the winter conditions from cold and dry to moist and mild. Conversely, Heilongjiang Province is landlocked and has a monsoon climate, which is drier and colder in winter, owing to northwest winds from Siberia.

This study also found a higher prevalence of chronic pain in women than in men. Physiological hormonal influences may underlie these differences in prevalence. Women have higher levels of oestrogen and lower levels of testosterone than men. Testosterone is a protective factor against pain (Athnaiel et al. 2023), while increased pain sensitivity is strongly associated with elevated estradiol levels (Hellman et al. 2021). In contrast, changes in hormone levels have been shown to induce pain sensitization (Athnaiel et al. 2023), but cyclic variations in oestrogen, progesterone, follicle‐stimulating hormone, and luteinising hormone levels are closely related to ovulation and menstruation in women. All these hormonal differences lower the pain threshold in women, making them more sensitive and susceptible to chronic pain.

Alcohol consumption can lower pain thresholds, cause pain, and increase the prevalence of cerebral palsy. Research has demonstrated that such pain includes “not only acutely enhanced pain associated with acute alcohol withdrawal and AUD (alcohol use disorder) but also chronic pain due to peripheral neuropathic effects of alcohol” (Robins et al. 2019; Monforte et al. 1995). Alcoholic neuropathy may develop with prolonged chronic alcohol consumption. Alcohol use no longer provides analgesia, and further drinking increases pain. Alcohol drinkers are vulnerable to this cycle, and chronic pain is a risk factor for alcohol relapse. Patients try to use alcohol to alleviate pain, but alcohol leads to nociception and the ingestion of more alcohol (Robins et al. 2019). Heavy consumption of alcoholic beverages decreases nutritional intake, resulting in deficiencies in various nutrients, such as proteins, minerals, and vitamins. Insufficient intake and conversion of calcium and vitamin D affect the dynamic balance between osteoblasts and osteoclasts of the skeleton, which can affect the skeleton and lead to related diseases (González‐Reimers et al. 2011). However, prolonged nutritional deficiency reduces synovial fluid secretion, leading to cartilage malnutrition and susceptibility to degenerative changes caused by minor external forces. Individuals who consume alcoholic beverages are more susceptible to trauma, which is correlated with chronic pain. The effects of alcohol on cortical functioning, including motor incoordination, amnesia, hypnosis, and ultimately unconsciousness (Harrison et al. 2017), can lead to unresponsiveness and confusion, which, in turn, triggers trauma and accidents (e.g., traffic accidents) (Martin et al. 2017).

This study suggests that the poorer the quality of sleep, the greater the risk that a participant will experience chronic pain. Those experiencing chronic pain are often caught in a vicious cycle—pain disrupts sleep, and short or restless sleep lowers pain thresholds and increases spontaneous pain. Sleep‐related endocrine factors, such as pineal melatonin, have anti‐inflammatory and analgesic effects. Disruptions in neurobiological systems or mediators with analgesic or nociceptive sensitization properties, such as the pineal melatonin system, hypothalamic–pituitary–adrenal axis, and immune system, increase the chances of experiencing chronic pain in individuals with poor sleep quality (Haack et al. 2020). However, the two sleep phases, REM and non‐REM sleep, alternate throughout the night in adults with normal sleep patterns, and the appearance of this regular cycle facilitates increased blood flow to the brain, protein synthesis, and systemic transport and metabolism of waste products, thereby reducing pain (Yuan et al. 2025).

A family history of pain is strongly associated with chronic pain. Genetic factors, particularly epigenetic inheritance, have profound effects on pain. At the cellular and molecular level, structural changes in chromatin caused by neurogenic and inflammatory pain injury have been shown to cause stable changes in gene expression and nerve function, potentially inducing a variety of symptoms, including abnormal pain, nociceptive hypersensitivity, anxiety, and depression (Descalzi et al. 2015). The family aggregation that pain exhibits may be related to shared eating, behavioral, and lifestyle habits. For example, some families do not pay attention to whether their children sit or stand straight. The statistically significant association between chronic pain and family history may also be related to families' material conditions and cultural backgrounds. Students from well‐off families can seek medical attention as soon as they experience chronic pain, and they may also purchase lumbar pads, chairs with the correct height, and specialised aids to protect their knees, while other students choose to put up with the pain or delay treatment due to financial considerations.

The frequency of ordering takeout food per week was another significant factor associated with chronic pain. Takeout meals are often heavy in oil and salt and lack vegetable and fruit content compared to other meals. Such dietary structure is highly likely to alter the composition of the gut microbiome. Previous studies have shown that the gut microbiome is critical for human health and disease. The composition of the human gut microbiome is influenced by factors such as host diet, which, in turn, affects host metabolism. Reduced gut microbial diversity is also associated with the development of chronic pain (Freidin et al. 2021; Kurilshikov et al. 2017; Luca et al. 2018; Janssen and Kersten 2017). High‐fat intake and lack of moderate exercise may lead to obesity, greatly increasing stress on the spine and all weight‐bearing joints throughout the body. An increase in total body fat has been shown to be strongly associated with pain (You et al. 2022). Many nursing students who ordered takeout meals did so because of the long and intense study hours and the lack of sufficient time to cook their own meals, highlighting their high psychological stress and heavy study load, along with their limited rest time to alleviate somatic fatigue and relieve chronic pain.

The prevalence of chronic pain increased with the nursing students' overall mean time per day spent using electronic devices. When using electronic devices, students are often in a forward‐flexed neck and seated position, exponentially increasing the bearing force on the skeletal joints from the perspective of physical force analysis, easily leading to aging of the joints and deformation of the intervertebral discs. In addition, work requirements or psychological addiction to the internet lead nursing students to use cell phones, keyboards, or mice in the same position for extended periods, greatly increasing their chances of chronic pain. Repeatedly using body parts, such as certain interphalangeal and wrist joints, can cause wear and tear, edema, ligament compression, and localised aseptic inflammation, leading to tenosynovitis or muscle strain in the hand and forearm (Chen et al. 2025; Özalp and Güven 2025; Alghadir et al. 2025).

Therefore, nursing students should adopt a healthy lifestyle. Individuals are advised to focus on achieving a healthy diet, such as consuming more fruits, vegetables, and mixed grains, while reducing the intake of takeout food, oil, salt, sugar, and alcoholic beverages to ensure a balanced intake of vitamins, proteins, lipids, carbohydrates, and other nutrients. Students would benefit from increased exercise and rest. During the break between classes, nursing students could relax their bodies and minds and improve their learning efficiency through short periods of meditation or stretching. They could be encouraged to reduce their dependence on computers and other electronic devices by switching from e‐books to paper materials and reducing the time spent on computer games and videos. Students could get closer to nature in their spare time, participate in sports, and socialise with their friends, ranging from running to travelling together. They could also be advised to learn ways to relieve their negative emotions and stress. For example, when running, the release of dopamine increases, which gives a sense of pleasure and relaxation and enhances physical fitness. Moreover, improving sleep quality is indispensable. Students can go to sleep and wake up on a schedule and avoid strenuous exercise and heavy mental activity before bedtime. Hot baths could also be recommended before bedtime. A gradual decline in the body temperature can promote sleep in the brain to produce sleepiness. Physical pain caused by muscle strain or joint wear and tear can benefit from muscle relaxation, massage, or hot compresses to ease discomfort. However, if symptoms are severe or recurrent, students should be encouraged to seek medical attention to reduce chronic pain through acupuncture, physical therapy, ointments, or surgery. Universities and colleges could set up relevant organizations responsible for managing students' diseases and health promotion and try to enhance students' awareness of chronic pain through teacher‐student exchanges or conference presentations to improve the nursing students' quality of life. The frequency of activities, such as physical exercise, can be increased in schools, which would not only help students change their sedentary habits and reduce lower back, neck, and shoulder pain but also increase emotional communication among students, reduce mental stress, and minimise the occurrence of headaches and other ailments that are closely related to psychological factors.

## 5 Limitations

The findings of this study have significant implications for developing health intervention programs to enhance nursing students' quality of life. However, several limitations should be considered when interpreting the results.

First, the cross‐sectional design precludes causal inferences. Second, the reliance on self‐reported data may introduce information bias. Specifically, sleep quality and academic stress were assessed using single global items rather than validated multidimensional instruments (e.g., PSQI), which could result in measurement error. However, such non‐differential error typically biases estimates toward the null, suggesting that the observed associations may be even stronger than reported. Third, although our definition of chronic pain strictly followed IASP guidelines, we lacked validated pain interference scales (e.g., Brief Pain Inventory) and objective assessments (e.g., physical examinations), which may affect the reported pain prevalence and functional impact. Fourth, the sampling strategy may have introduced selection and non‐coverage biases. The use of cluster sampling excluded students on internships or leave, and students who never clicked the survey link were systematically excluded. Additionally, restricting the sample to first‐year students limits generalizability to more senior populations, though this design choice strengthened internal validity by controlling for professional exposure time. The unequal gender distribution, while reflecting the current demographic of the nursing profession in China, may also have influenced statistical results.

## 6 Conclusion

In summary, chronic pain is commonly reported by nursing students. Early assessment and intervention at different pain sites are necessary to reduce the potential negative effects of pain. Variables associated with an increased risk of chronic pain among nursing students included perceived academic stress, alcohol consumption, smoking, frequency of ordering takeout food, time spent on electronic devices per day, and sleep quality. Interventions that address these specific factors could improve students' chronic pain and enhance their quality of life.

## 7 Implications for the Profession and/or Patient Care

This study provides important empirical evidence for improving the overall well‐being of nursing students and optimising the nursing education system. The findings offer new insights for educational institutions and relevant administrators to implement better management practices.

First, the study revealed a high prevalence of chronic pain among nursing students and identified numerous potential influencing factors, underscoring the necessity of integrating systematic occupational health education into the curriculum. Through theoretical instruction or simulated exercises, students can acquire techniques for self‐correction of poor posture before entering clinical internships, thereby reducing the risk of occupational musculoskeletal disorders at their source. By intervening in these modifiable factors—such as promoting lifestyle changes, reducing the time spent using mobile phones and other electronic devices, or cultivating healthy sleep habits—we can effectively reduce the prevalence of chronic pain and enhance the quality of life of nursing students.

Second, the study found that factors such as alcohol consumption and electronic device usage time were associated with chronic pain in nursing students. Schools and clinical internship bases should establish regular health monitoring mechanisms to facilitate early identification of and intervention for high‐risk students. Furthermore, institutions can offer targeted physical activity clubs, such as yoga and core strength training, or provide psychological counselling services to alleviate student stress. These initiatives can help students strengthen their physical fitness and improve their ability to cope with chronic pain.

Finally, these findings also call upon educational administrators to re‐evaluate the support systems within clinical internship environments. We recommend that clinical instructors enhance their guidance on students' ergonomic practices and advocate for a superior internship culture. This approach not only effectively prevents chronic pain among nursing students and improves their quality of life during both academic and internship periods but also cultivates nursing professionals with sustainable development capabilities, ultimately contributing to the provision of higher quality care for patients.

## Author Contributions

Conceptualization: Siqi Liu, Yang Fu, Lei Shi. Data curation: Yujin Xie, Lei Cao. Formal analysis: Xiwei Chen, Zhengxi Chen. Funding acquisition: Yang Fu, Lei Shi. Investigation: Siqi Liu, Yang Fu. Methodology: Lei Shi. Project administration: Mei Yin. Resources: Yang Fu, Lei Shi. Software: Xiwei Chen, Lei Shi, Zhengxi Chen. Supervision: Yang Fu, Lei Shi, Yue Qin. Validation: Yang Fu, Lei Shi. Visualization: Xiwei Chen, Lei Shi. Writing – original draft: Siqi Liu. Writing‐review and editing: Siqi Liu, Yang Fu, Lei Shi.

## Funding

School of Humanities, Harbin Medical University: HMURW20210203. Guangdong Basic and Applied Basic Research Foundation: 2023A1515010902. Guangdong Province Youth Research Co‐construction Project: 2023GJ064. Youth Science and Technology Talent Development Program of Guangdong Association for Science and Technology: SKXRC2025206.

## Conflicts of Interest

The authors declare no conflicts of interest.

## Supporting information


**Data S1:** nop270685‐sup‐0001‐Appendix.docx.

## Data Availability

The data that support the findings of this study are available from Yang Fu and Lei Shi. *Data Storage Location*: All data collected in this study is stored on offline office computers located in the offices of Yang Fu and Lei Shi. These computers are not connected to any network, ensuring an additional layer of physical isolation from external access. *Data Retention Period*: In accordance with institutional research data policies, the data will be retained for a period of 10 years following the completion of the study. *Access Control*: Access to the data is strictly limited to the two investigators mentioned above (Yang Fu and Lei Shi). The database files are protected with password encryption, and only the designated computers used by these two researchers are authorised to access the data. No other individuals have physical or remote access to the stored information. *Data Protection and Encryption*: In addition to password protection at the database level, the offline nature of the storage computers eliminates risks associated with network‐based breaches. Physical security measures are also in place to restrict entry to the offices where these computers are located.
