# Supplementary material for: Prevalence of Chronic Pain and Associated Factors Among Nursing Students in Heilongjiang Province, China: A Cross‐Sectional Study
Source: Nurs Open. 2026 Aug 13;13(8):e70685. doi: 10.1002/nop2.70685 (PMC13472539; doi:10.1002/nop2.70685)
Supplement: Supplementary file 1 [file NOP2-13-e70685-s001.docx]

**Recruitment Channels and Timeline**
The survey was conducted from June 13 to June 15, 2023, among college students at a nursing vocational college in Heilongjiang, China. A link to the questionnaire was distributed through the university's academic management system and class WeChat groups across various departments. The questionnaire was completed anonymously, with each student allowed to submit only one response. The system identified duplicate submissions by tracking IP addresses and device information.

**Invitation Wording**
Potential participants were recruited using the following invitation:

Dear Students,
We are conducting this survey to better understand the lifestyle habits of college students. The questionnaire is anonymous, and your responses will not be disclosed to any individual or organization beyond yourself. Please answer based on your actual circumstances. Your participation will provide valuable insights, and we sincerely appreciate your support and cooperation!

Research Group on Nursing Students' Lifestyle Habits and Pain
This invitation was distributed along with the questionnaire link, and no material or financial incentives were offered.

**Participation, Consent, and Completion Rates**
During the recruitment period:

**View Rate**: A total of 1,825 invited individuals clicked and viewed the questionnaire link (i.e., opened the first page of the survey).

**Consent Rate**: All viewers clicked "agree to participate" and proceeded with the survey, resulting in a 100% consent rate.

**Completion Rate**: All 1,825 individuals who consented completed all survey questions, yielding a 100% completion rate.

**Final Valid Sample**: During data cleaning, 23 questionnaires were excluded based on predefined criteria: (1) poor quality responses (i.e., incomplete items, inconsistent answers and those which were completed within 100 seconds), (2) the inability to understand the meaning of the written text, and (3) a diagnosis of a congenital musculoskeletal or neurological disorder. This resulted in 1,802 valid questionnaires, representing a valid response rate of 98.7%.

**Incentives**
No monetary rewards, gifts, or academic credits were provided for participation, which was entirely voluntary.
